# Study of association and molecular analysis of human papillomavirus in breast cancer of Indian patients: Clinical and prognostic implication

**DOI:** 10.1371/journal.pone.0172760

**Published:** 2017-02-28

**Authors:** Saimul Islam, Hemantika Dasgupta, Anirban Roychowdhury, Rittwika Bhattacharya, Nupur Mukherjee, Anup Roy, Gautam Kumar Mandal, Neyaz Alam, Jaydip Biswas, Shyamsundar Mandal, Susanta Roychoudhury, Chinmay Kumar Panda

**Affiliations:** 1 Department of Oncogene Regulation, Chittaranjan National Cancer Institute, Kolkata, West Bengal, India; 2 Department of Pathology, North Bengal Medical College and hospital, Sushruta Nagar, Darjeeling, West Bengal, India; 3 Department of pathology, Chittaranjan National Cancer Institute, Kolkata, West Bengal, India; 4 Department of Surgical Oncology, Chittaranjan National Cancer Institute37, Kolkata, West Bengal, India; 5 Department of Epidemiology & Biostatistics, Chittaranjan National Cancer Institute, Kolkata, West Bengal, India; 6 Saroj Gupta Cancer Centre and Research Institute, Thakurpukur, Kolkata, India; University of Navarra, SPAIN

## Abstract

**Objectives:**

Human papillomavirus (HPV) causes tumors primarily Cervical cancer. Recently, inconsistent reports came up in Breast cancer (BC) too. In India, despite treatment 70,218 BC patients die each year. So, we explored the association of HPV, if any, with BC prognosis in Indian pre-therapeutic (PT) and Neo-adjuvant chemotherapy (NACT) patients with subsequent analysis of HPV profile.

**Methods:**

HPV prevalence was checked and analysis of physical status, copy number, genome variation, promoter methylation and expression (mRNA and protein) of the prevalent subtype was done.

**Results:**

High prevalence of HPV was observed in both PT (64.0%) and NACT (71.0%) cases with significant association with younger (20–45 yrs) PT patients. Interestingly, HPV infection was significantly increased from adjacent normal breast (9.5%, 2/21), fibro adenomas (30%, 3/10) to tumors (64.8%, 203/313) samples. In both PT and NACT cases, HPV16 was the most prevalent subtype (69.0%) followed by HPV18 and HPV33. Survival analysis illustrated hrHPV infected PT patients had worst prognosis. So, detailed analysis of HPV16 profile was done which showed Europian-G350 as the most frequent HPV16 variant along with high rate of integration. Moreover, low copy number and hyper-methylation of P97 early promoter were concordant with low HPV16 E6 and E7 mRNA and protein expression. Notably, four novel variations (KT020838, KT020840, KT020841 and KT020839) in the LCR region and two (KT020836 and KT020837) in the E6 region were identified for the first time along with two novel E6^E7*I (KU199314) and E6^E7*II (KU199315) fusion transcript variants.

**Conclusion:**

Thus, significant association of hrHPV with prognosis of Indian BC patients led to additional investigation of HPV16 profile. Outcomes indicated a plausible role of HPV in Indian BC patients.

## Introduction

Human papillomavirus (HPV) is a DNA virus having a 9.2Kb genome. The high-risk (hr) subtypes were frequently associated with different cancers, primarily cervical cancer (CACX) and Head and neck squamous cell carcinoma (HNSCC) [1]. Among the subtypes of hrHPV, HPV16 was the most prevalent subtype in these tumors [2]. It is well known that HPV16 mainly infects the basal epithelial stem cell through break of stratified epithelial layer [3]. The transformation HPV depends on expression of E6/E7 oncoproteins which contributes to the process of carcinogenesis by increasing cellular proliferation leading to more genomic instability and inhibition of apoptosis [4]. The expression of both the oncoproteins is controlled by viral protein E2, which often gets abrogated due to viral integration in the host genome through the E2 region [5]. In addition, expression of E6 and E7 has been regulated by the long control region (LCR) harboring different transcription factors binding sites along with the viral copy number variation though the activity of P97 promoter and enhancer [6, 7]. The activity of this promoter and enhancer were further controlled by methylation though host DNA Methyltransferase enzymes [8]. Moreover, it was also reported that RNA splicing may control the expression of these oncoproteins as well [9].

In recent times, several worldwide reports have associated HPV with Breast cancer (BC) while other investigators have strongly negated it. A current meta-analysis encompassing 21 different studies had also revealed this inconsistency in prevalence of high risk HPV16 (ranging from 3% to 92%), HPV18 (2% to upto 100%) and HPV33 (4% to 70%) [10]. HPV16 infection (40%, 4/10) was also reported in neo-adjuvant chemotherapy treated (NACT) samples [11]. In addition, HPV was also detected in normal (1–29%) and benign (13.7–55%) breast tissues[12–14]. It was further reported that the E6 and E7 oncoproteins of HPV16 could immortalize human mammary epithelial cells indicating their importance in cellular transformation [15]. Previous studies reported frequent integration of HPV16 in BC patients, whereas, contradictory reports of low viral copy number and reduced expression of HPV16 oncoproteins E7 (protein) has also been found [14, 16]. Interestingly, BC patients with HPV16 infection have been reported to show better prognosis [17]. So, association of HPV with BC still remains controversial.

On the other hand, in India, where 144,937 BC cases were newly diagnosed and 70,218 BC patients die per year in spite of therapy, variable frequencies (from negative to up to 26.5%) of HPV infection in BC have been found [18, 19]. But till now no in depth study to analyze the association of HPV with BC has been carried out.

Thus, in the present study, attempts have been made to analyze the association of HPV, if any, in BC of Indian patients. At first, we analyzed the prevalence of HPV in 272 pre-therapeutic and 41 NACT BC of Indian patients followed by prevalence of high risk HPV16, 18 and 33. Our study showed high frequency (63.9%-71%) of HPV infection in the both pre-therapeutic and NACT samples with highest prevalence of HPV16. PT patients with hrHPV infection showed worst prognosis as well. So, further analyses were done to decipher the genetic and epigenetic status of HPV16. Finally, integrated genome, P97 promoter methylation, low viral copy number along with reduced expression (mRNA/protein) of its oncoproteins E6/E7 were the attributes of HPV16, frequently encountered in Indian BC patients.

## Materials and methods

### Patient population, tumor tissues and cell lines

A total of 272 freshly operated pre-therapeutic and 41 neo-adjuvant chemotherapy treated breast tumor specimens along with 21 adjacent normal tissues, 10 fibro adenomas and 7 benign phyllode were collected from Chittaranjan National Cancer Institute(CNCI), Kolkata, India, after appropriate approval of Institutional ethical committee and written consent from individuals. After surgery, some portion of the operated specimen was collected by the surgeons in sterile pots in the operating room. These were then cut into pieces by sterile surgical blades. One part of the tissues were stored in 10% formalin for immunohistochemistry (IHC) analysis and the remaining part stored at -80°C in isolated rack until further use. In addition, standardized precautions were taken to avoid contamination during tissue preparation. For RNA analysis some of the pre-therapeutic samples (N = 10) were kept in RNAlater^®^ (Ambion, USA) solution which is separately stored. Among these samples 56.8% (178/313) patients were below the average age of 45.6 years and 43.1% (135/313) were above the average age (S1 Table). As samples were collected before and after therapy, we have designated as pre-therapeutic and chemotherapy treated samples (NACT). Breast cancer cell line MCF7 and cervical cancer cell line SiHa were obtained from National Centre for Cell Science, Pune, India (S1 Fig).

### Isolation of DNA from tumor samples

The contaminant normal cells in the breast lesions were removed by micro-dissection procedure [20] to enrich (>80%) tumor cells for isolation of high-molecular weight DNA by proteinase-K digestion followed by phenol-chloroform extraction [21]. Separate sterile work place was used and every feasible precaution was additionally taken so that no contaminations from surroundings occur.

### HPV detection and sub-typing

The presence of HPV in BC was detected by PCR using primers (MY09 and MY11) from the consensus L1 region [22]. Typing of HPV-16/18/33 in the L1-positive samples was done by PCR using specific primers (S2 Table) The HPV16, HPV18 and HPV33 plasmids were used as positive control for the respective cases [22, 23]. Further validation was done by Southern hybridization using ^32^P-labeled HPV16 and HPV18 type-specific probes [24]. All PCR reactions were done in clean restricted work surface where other laboratory works were not performed.

### Determination of HPV16 physical status

The physical status of HPV16 genome was determined by multiplex PCR using three different primer sets (S2 Table) and the ratio of E2/E6 copy numbers was calculated as described previously [25]. For episomal and integration control, HPV16 plasmid and SiHa (HPV16 positive) were used respectively. Further validation was done in 23 randomly selected samples by real time PCR using Power SYBR Green (Applied Biosystems, USA) with the same primer sets and controls [26].

### Sequencing analysis of HPV16 LCR, E6 and E7 regions

Using two set of primers (S2 Table), LCR and E6-E7 regions were sequenced in both sense and antisense directions with a BigDye Terminator Cycle Sequencing 3.1 Kit (Applied Biosystems, USA) according to the manufacturer's instructions in a 3130xl Genetic Analyzer (Applied Biosystems, USA) [25]. Designation of phylogenetic clusters of HPV16 E6 region was done according to Yamada et al [27].

### Methylation analysis of LCR

The methylation status of the p97 promoter and enhancer region was analyzed by methylation sensitive restriction enzyme analysis (MSRA) using enzymes HpaII and HhaI for digestion of P97 promoter and enhancer respectively [28]. The 445 bp fragment of β-3A adaptin gene (K1) and 229 bp fragment of RARβ2 (K2) were used as digestion and integrity controls [29].

### Estimation of HPV16 copy number

The copy numbers of HPV16 in the samples were determined by TaqMan absolute Real-Time PCR method using specific probe (S2 Table) as described previously [30].

### Quantification of HPV16 E6 and E7 transcripts

Total RNA was extracted from HPV16 positive breast tumors and the two cell lines MCF7 and SiHa [31]. Relative quantification of E6/E7 expression was performed using a power SYBR-green assay (Applied Biosystems, USA) with β2-microglobulin as endogenous control [25]. The RT-PCR products were electrophoresed in 2% agarose gel for detection of different splice products of E6/E7 mRNA and were further characterized by Sanger sequencing using specific primers (S2 Table).

### Detection of HPV16 E6 and E7 proteins expression

The goat polyclonal antibody for E6 (sc-1584) and mouse monoclonal antibody for E7 (sc-6981) were used at a dilution of 1:200. The HRP conjugated rabbit anti-goat IgG (sc- 2768) and goat anti-mouse IgG (sc-2005) were used at a dilution of 1:500 as secondary antibody in respective cases, followed by scoring as mentioned previously [30]. The antibodies were purchased from M/s Santa Cruz Biotechnology, CA, USA.

### Bio-informatics analysis for E6 and E7 mRNA and protein stability

Transcription factor prediction was done using on line server *Alibaba 2*.*1* [32]. The protein sequence and half-life of different splice variants of E6/E7 transcript were predicted by transcription-translation tool and *ProtLifePred* wave server respectively [33]. The stability of E6 variants in both mRNA and protein were predicted by *mFOLD* and *I-Mutant 2*.*0* followed by validation in *Proven* wave server [34–36].

### Statistical analysis

Chi-square analysis was used to determine association between HPV profiles and different clinicopathological parameter of tumors using Epi Info 6.04. Survival analysis (upto 5 years) was performed by Kaplan–Meier method using SPSS 10.0. P-value ≤0.05 was considered statistically significant in all analysis.

## Results

### Prevalence of HPV in breast tissues

**Fig 1:**
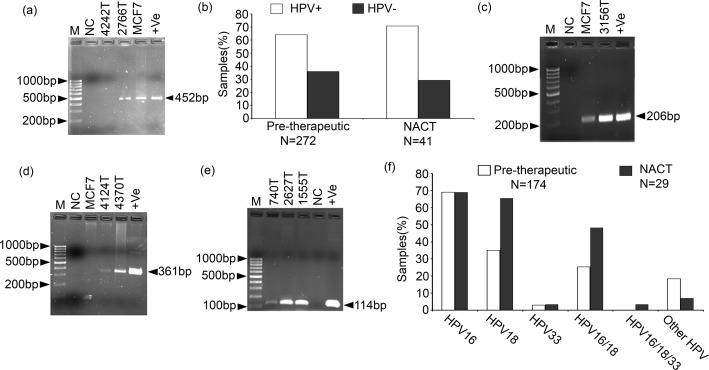
Determination of HPV prevalence in primary breast tumors and breast cancer cell line MCF7. **(a)** Representative agarose gel showing positive HPV infection in pre-therapeutic sample 2766T and MCF7 cell line while, neo-adjuvant chemotherapy treated (NACT)sample 4242T is HPV negative. +Ve means Positive control having HPV16 plasmid. **(b)** Frequency of HPV infection in BC samples. Samples were identified as positive when PCR bands were seen with respective subtype specific primers. Representative agarose gel of **(c)** HPV16 **(d)** HPV18 **(e)** HPV33 detection PCR. [M: 100bp marker, NC represent Negative control with no DNA, +Ve represent Positive control having HPV16, HPV18 and HPV33 plasmid in their respective subtype detection gels] **(f)** Distribution of different genotypes of HPV in different type of BC samples.

Using primers MY09/MY11, total HPV prevalence was detected in 63.9% (174 / 272) in pre-therapeutic and 71.0% (29/41) in NACT BC samples (Fig 1A and 1B). Among the HPV positive pre-therapeutic BC samples, the prevalence of HPV16, HPV18 and HPV33 were in the following order: 69.0% (120/174) > 35.0% (61/174) >2.9% (5/174) respectively (Fig 1C, 1D, 1E & 1F and S2A & S2B Fig). The co-infection of HPV16 and HPV18 was seen in 25.2% (44/174) samples. About 18.3% (32/174) samples showed other than HPV16, HPV18 and HPV33 infection. Similar trend of the HPV subtypes infection was seen in NACT BC samples with 69.0% (20/29) HPV16, 65.0% (19/29) HPV18, 3.4% (1/29) HPV33 and 6.9% (2/19) other HPV subtypes (Fig 1F). About 48.2% (14/29) samples showed co-infection of HPV16 and HPV18, and 5% (1/19) samples showed co-infection of HPV16, HPV18 and HPV33. The breast cancer cell line MCF7 showed HPV16 infection (Fig 1A, 1C & 1D and S2A & S2B Fig). However, only 9.5% (2/21) adjacent normal breast tissues showed HPV infection although their tumor tissues were HPV positive. In addition, fibro adenomas and phyllode samples showed 30% (3/10) and 71.5% (5/7) HPV infection. Surprisingly, the HPV infection was significantly (p = 0.001) increased during progression from normal to tumor (Table 1). Interestingly, significant high HPV infection was seen within average value of age of onset (45 yrs) of pre-therapeutic BC samples (p = 0.02) (S3 Table). However, no such correlation was seen in NACT samples. On the other hand, no significant correlation of total HPV/different HPV subtypes infection were seen in different clinicopathological parameters in univariate and multivariate analysis of pre-therapeutic BC samples (S3 Table). Similar trend was also seen in NACT samples.

**Table 1:** Comparative HPV infection in adjacent normal breast, fibro adenomas, benign phyllode and breast tumors.

Sample Type	HPV positive (%)	HPV negative (%)	P value
Normal n = 21	2(9.5)	19(90.5)	0.001*
Fibro adenomas n = 10	3(30.0)	7(70.0)	
Benign phyllode n = 7	5(71.5)	2(28.5)	
Breast cancer n = 213	203(64.8)	110(35.2)	

* indicate significant correlation (P≤0.05).

**Fig 2:**
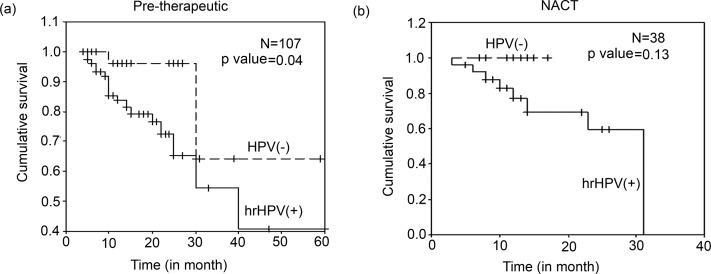
Kaplan–Meier 5-year survival probability curves with cumulative survival of breast cancer patients (BC) based on hrHPV status. Survival probability of BC patients in **(a)** pre therapy group (p = 0.04) and **(b)** neo-adjuvant chemotherapy treated (NACT) group (p = 0.13). [N: total number of samples included in this study. hrHPV(+) indicates infection by either HPV16, HPV18 or HPV33].

The Kaplan–Meier survival analysis showed significantly (p = 0.04) poor survival of hrHPV positive than overall HPV negative pre-therapeutic patients (Fig 2A). However, no such significance has been observed in NACT cases (Fig 2B). Similar trend in survival was seen when overall HPV positive pre-therapeutic and NACT samples were compared with their respective HPV negative group, though it was not statistically significant (p = 0.05) (S3A and S3B Fig). As hrHPV infection showed significant correlation with worst BC prognosis, so detailed genetic (physical status, copy number variation and sequence variation) and epigenetic (P97 promoter and enhancer methylation) profiles of HPV16 were performed in BC samples.

### Physical status of HPV16 in BC

**Fig 3:**
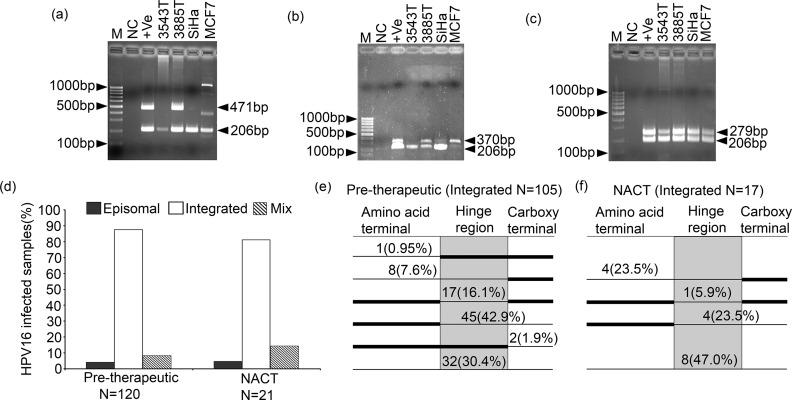
Analysis of HPV16 physical status in breast tumor samples and MCF7 breast cancer cell line. Representative agarose gel of physical status of HPV16 genome at **(a)** E2A region. **(b)** E2B region and **(c)** E2C region **(d)** Histogram represent significant high frequency of integrated viral genome in both pre-therapeutic and NACT samples (p≤0.01). Frequency of integration at three regions of the E2 gene in **(e)** pre-therapeutic cases and **(f)** NACT samples. [Here M: 100bp marker, NC represent Negative control with no DNA, +Ve represent Episomal control where HPV16 plasmid was used. SiHa is the HPV16 positive Cervical cancer cell line used as Integration control for E2A and E2B region while Episomal control for E2C region]

In pre-therapeutic BC samples, significantly high HPV16 integration (87.5%, 105/120) was seen compared to the episomal form (4.2%, 5/120) (p = 0.01) (Fig 3A, 3B, 3C and 3D). Both episomal and integrated forms i.e mixed form, was evident in 8.3% (9/120) samples (Fig 3D). Similar trend was seen in NACT samples with 81.0% (17/21) integrated form, 4.8% (1/21) episomal form and 14.3% (3/21) mixed form (Fig 3D). Significant concordance (p = 0.75) was evident between the multiplex PCR and the Real Time PCR method of analysis of HPV16 physical status (data not shown). Interestingly, HPV16 integration was seen mainly in the hinge region of E2 in 97.0% (102/105) pre-therapeutic and 100.0% (17/17) NACT samples (Fig 3E and 3F). In MCF7, HPV16 was present in an episomal form with insertion/ deletion in the N-terminal region due to the presence of two altered PCR products of E2A primer (Fig 3A, 3B and 3C). The HPV16 physical status showed no significant correlation with different clinico-pathological parameters due to high prevalence of the integrated form (data not shown).

### Sequence variation analysis of LCR and E6-E7 region of HPV16 in BC

**Fig 4:**
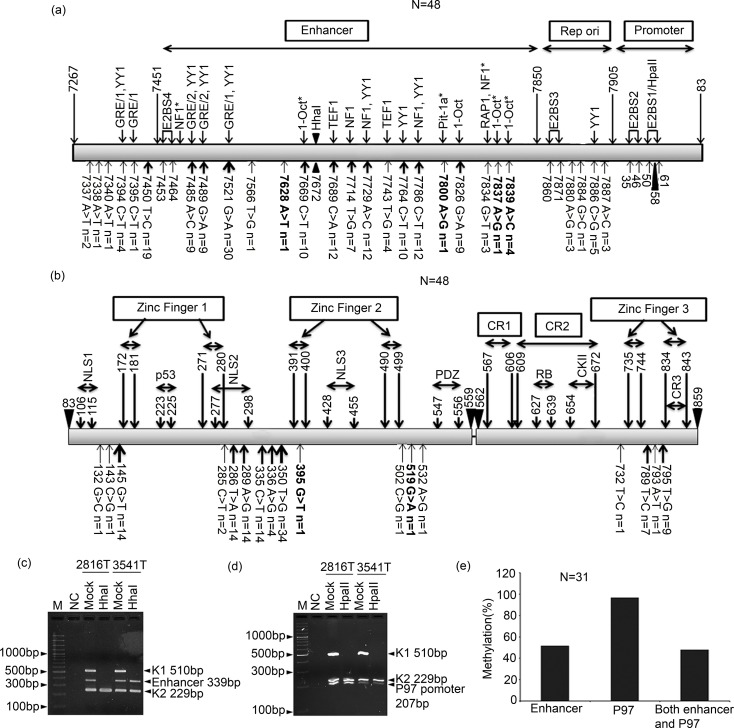
Sequence variation of LCR, E6 and E7 region and methylation status of LCR of HPV16 in pre-therapeutic breast tumor cases. Schematic representation of identified sequence variations in **(a)** the LCR region and **(b)** the E6 and E7 gene. Here, bold lettered variants were novel ones. Upper head arrow thickness indicate the frequency of the given variation where “N” represent number of samples. In LCR, lower head arrows indicates binding of the transcription factors while in E6 and E7 gene it highlighted different protein domains. Methylation sites in the LCR were also marked (Bold triangle). ‘*’ indicates transcription factors predicted by Alibaba 2.1 TF Binding Prediction software. Representative agarose gel showing methylation status at **(c)** the enhancer region **(d)** the P97 promoter region. **(e)** Histogram showed significant high frequency methylation in P97 promoter than enhancer region (p = 0.004). [M: 1000bp marker, NC represent Negative control with no DNA, K1 used as digestion control, K2 used as DNA integrity control, Mock: mock digestion without enzyme, HhaI: DNA digested with HhaI enzyme, HapII: DNA digested with HpaII enzyme].

The total sequence variation was seen in 70.8% (34/48) pre-therapeutic BC samples with 21 variant in the LCR region, 11 variant at E6 and 3 variant at E7 regions (Fig 4A & 4B). In LCR region, scattered sequence variation was evident with high 62.5% (30/48) 7521G>A transition in the samples (Fig 4A). The majority of sequence variations were overlapped with different transcription factors binding sites like YY1, GRE1, Oct-1 etc (Fig 4A). Five samples showed 7886C>G transversion in the replication origin containing YY1 repressor binding site. Four novel variants at 7628A>T (KT020838), 7800A>G(KT020840), 7837A>G(KT020841) and 7839A>C (KT020839) were identified in 7 samples (Fig 4A). The variant at 7839A>C (KT020839) was seen in four samples and other variants were seen in one sample each (Fig 4A). These novel variants overlapped with Oct-1 and Pit-1a transcription factors binding sites (Fig 4A).

In E6 region, the sequence variations were evident in flanking of the two zinc finger domains with high frequency (70.0%, 34/48) of 350T>G transversion followed by 18.7% (9/48) variation at 286T>A and 289A>G sites (Fig 4B). Two novel variations were identified at 395G>T (KT020836)(2.0%, 1/48) and 519G>A (KT020837) (2.0%, 1/48) sites (Fig 4B). Majority 54.5% (6/11) of the variations affected biological function of E6 protein. In the E7 region, the sequence variation was seen mainly in the zinc finger 3 domain with 19.0% (9/48) at 795T>G and 14.5%(7/48) at 789T>C sites (Fig 4B). Seven samples showed both 795T>G and 789T>C variations. In addition, one sample showed variation in flanking of the zinc finger domain at 732T>C. After compilation of the sequence variation at E6 and E7 regions, it was evident that the European lineage with T350G variant (E-G350) (1 of which was E-G350 G519A) was frequent 39.6% (19/48) followed by 29.0% (14/48) of European prototype (Ep), 19.0% (9/48) of North American 1 (NA1), 8.0% (4/48) of North American 1 variant (NA1A336G), 2.0% (1/48) of Asian American variant (AA G395T) and 2.0% (1/48) of African1 (AF1).

### Analysis of LCR region methylation of HPV16

Significant high methylation (96.7%, 30/31) was seen in P97 promoter region than the enhancer region (51.6%, 16/31) (p = 0.004) (Fig 4C, 4D & 4E). The methylation in both enhancer and promoter regions were seen in 48.0% (15/31) of the samples (Fig 4E). The overall LCR region methylation showed no association with different clinicopathological parameters (data not shown). The genetic variations seen in the LCR region (Fig 4A) were not overlapped with the P97 promoter and enhancer methylation sites (nt58 and nt7672).

### Analysis of copy number of HPV16 in pre-therapeutic BC

The wide variation of HPV16 copy number (0.58–1044.6 copies/ 50ng gDNA) with the median value of 9.3 copies/ 50ng gDNA was observed in BC (S4A Table). There was gradual increase in HPV16 copy numbers with progressive cytological severity, stage and nodes at pathology (S4B Table). Among the different HPV16 lineages, the E-G350 lineage showed high copy number (S4B Table).

### Expression analysis E6 and E7 of HPV16

#### mRNA

**Fig 5:**
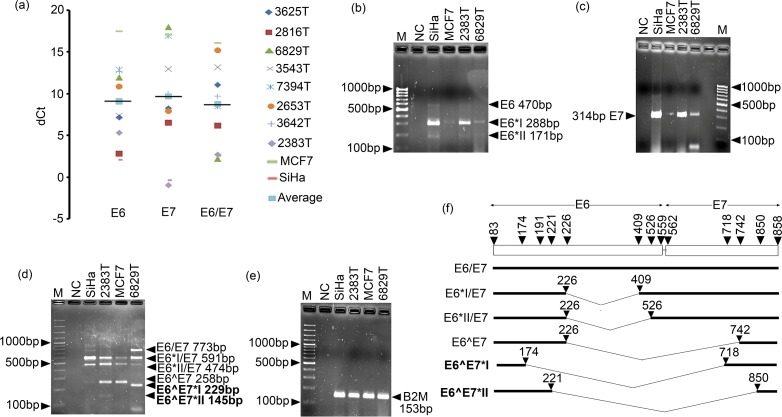
Detection and quantification of E6 and E7 mRNA of HPV16 in pre-therapeutic breast tumor samples and MCF7 breast cancer cell line. **(a)** The dot plot showed wide variation of dCt value of E6, E7 and full length E6/E7 mRNA expression. High dCt value indicates low expression and vice versa. Here, SiHa cell line was used as positive control for E6 and E7 expression. Bold horizontal line represent average dCt value. **(b)** Representative agarose gel of post real time PCR of E6 showed different splice forms of E6. **(c)** Agarose gel represents post real time PCR of E7. **(d)** Representative agarose gel of post real time PCR showed full lenth E6/E7 mRNA with its dfferent splice forms. Bold lettered represent novel splice form. **(e)** Representative gel showed post real time PCR of β2-microglobulin used as endogenous control gene. **(f)** Schematic diagram of different splice forms of E6 and E6-E7 transcripts. Bold line represent exon and V- shaped thin line represents intron. Nucleotide position in HPV16 genome was also indicated. Number of left and right arm represent splicing donar and acceptor sites respectively. [Here M: 1000bp marker; NC represent Negative control with no cDNA].

There were wide variations in expressions of E6, E7 and E6/E7 with mean value of 9.03, 9.6 and 8.7 respectively in different BC samples and the cell lines MCF7 and SiHa (Fig 5A–5E). Different splice variants of E6 and E6/E7 such as E6*I, E6*II, E6*I/E7, E6*II/E7 and E6^E7, were seen in different BC samples (Fig 5A & 5F and S4A & S4B Fig). Among the splice variants, E6*I/E7 transcript was the most prevalent (8/8). Two novel fusion transcripts E6^E7*I and E6^E7*II in two samples were sequenced and registered in Gene Bank with accession numbers KU199314 and KU199315 respectively (Fig 5F and S4C & S4D Fig). The bio-informatics analysis predicted that the novel E6 variant 519G>A (KT020836) seen in E-G350 lineage decreased the stability of both E6/E7 and E6*I/E7 mRNAs, whereas another novel E6 variant 395G>T (KT020837) seen in AA lineage decreased stability of E6/E7 mRNA only. In addition, low stability of two novel transcripts E6^E7*I and E6^E7*II were predicted than full length E6/E7 transcript using mFOLD wave server (data not shown). Moreover, transcription—translation tool and ProtLifePread server predicted that only E6^E7*II transcript could produce E6/E7 fusion protein with comparable half-life of E6^E7 transcript (data not shown).

#### Protein

**Fig 6:**
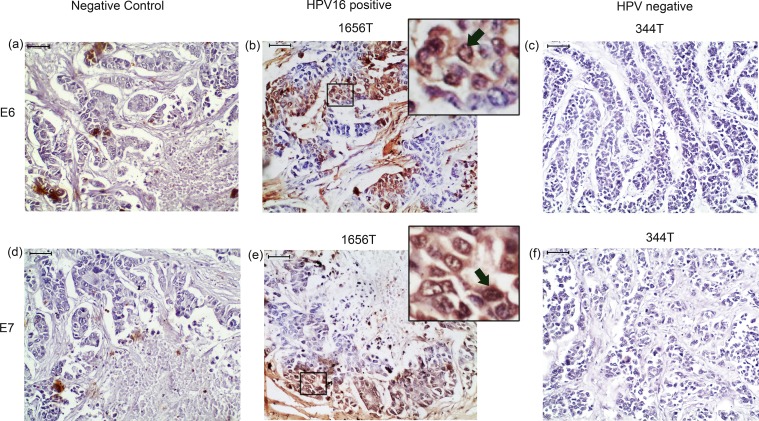
Immunohistochemical detection of E6 and E7 protein of HPV16 in pre-therapeutic breast tumor tissues. **(b) & (e)** Representative immunohistochemical staining of E6 and E7 in HPV16 positive samples. **(c) & (f)** Representative immunohistochemical staining in HPV negative sample **(a) & (d)** Immunohistochemical staining with out primary antibody represented Negative control (NC). [Magnification of tissue samples is 20X and for inset, magnification is 40X, Scale bars = 50 μm].

In immunohistochemical analysis, nuclear expression of E6 and E7 was seen in the HPV16 positive samples (Fig 6A–6F). About 53.3% (16/30) samples showed low E6 and E7 protein expression. The protein expression of both E6 and E7 showed concordance with respective mRNA expression (S5 Table). The bio-informatics analysis (I-Mutant 2.0 server) predicted that the novel E6 variant of 519 G>A of E-G350 lineage might have low protein stability than another novel E6 variant 395G>T in AA lineage with increased stability (data not shown).

## Discussion

The primary aim of the study was to understand the association of HPV infection with BC. As hrHPV infection showed statistically significant correlation with worst prognosis of BC patients so, further its genetic and epigenetic landscape of the most prevalent subtype (HPV16) was analyzed. At first, prevalence of HPV was analyzed in (Eastern) Indian BC patients (n = 313) and subsequently comprehensive analysis of HPV16 profile was performed. To the best of our knowledge such comprehensive analysis was done for the first time among Indian BC patients.

Our results showed frequent infection (63.9–71.0%) of HPV in pre-therapeutic and NACT BC samples, indicating plausible importance of HPV infection in development of this disease irrespective of therapy. The high HPV infection may be due to poor hygienic condition and malnutrition of patients together with ethnicity[19, 37]. Among the HPV positive samples, the prevalence of HPV16 was high (69.0%) followed by HPV18 (35.0%) and HPV33 (2.9%). For HPV detection, the gold-standard L1 consensus primers MY09/11 primers was used which can detect up to 40 mucosal HPV genotypes, including the most prevalent high risk (HPV 16 & 18) as well as the low risk (HPV 6 & 11) types [38]. Every precaution was taken to prevent any possible risk of contamination of PCR reactions [39]. Furthermore, the result was validated using Southern blot.

Similar to our data, high frequency (74.0%) of HPV infection was previously reported though there HPV33 was found to be the most prevalent subtype [40]. In contrast, investigators also informed moderate (21.0–48.0%), low (1.6–13.3%) or absence of HPV infection in BC with varied prevalence of HPV16 or HPV18 subtypes [10]. This ambiguity in frequency of HPV infection in BC might be due to differences in etiological factors, ethnicity and analysis methods [37]. Moreover, all necessary precautions were taken strictly while carrying out all the PCR reactions used for prevalence analysis (see Materials and Methods).

The presence of HPV in normal and benign tumor and sharp increase in malignant breast tumors indicates its pathological importance in breast cancer. Similar trend of HPV infection from normal to benign and tumor was also reported by Lawson et al [14]. Consistent with our data, recent meta-analysis showed that HPV infection was four fold increased in BC compared with normal breast tissues [41]. Similar to Lawson and Bae et al reports, in our study also high HPV infection (p = 0.02) was found in lower age group (≤ 45yrs) patients, which suggests that HPV infection, may aid in reducing the age of tumor onset [12]. Like earlier reports, no significant correlation of HPV infection was found with clinico-pathological parameters including, stage, grade, lymph node, age of onset and parity [17, 42, 43]. In addition, poor prognosis was found in hrHPV infected pre-therapeutic patients when compared with other groups (p = 0.04). Interestingly, no significant association with prognosis was seen when total HPV positive samples were considered similar to previous studies where no association of HPV-positive BC patients with clinical outcome was reported [11, 17]. It appears that BC patients with low-risk HPV infection may be at lesser risk unless they are co-infected or solely infected with hrHPV types though it is known that differences may occur due to ethnicity and sample size [37].

In HPV16 positive samples, HPV16 was integrated frequently (87.5%) in the host genome by disrupting the hinge region (E2B) of the E2 gene with gradual increase with stage. Such observation clearly indicates rise in virulence of HPV during progression of the tumor. Though frequent integration was seen previously in BC [11, 16] but only such disruption of E2B was reported in cervical carcinoma [44]. Presence of two altered PCR products of E2A region in MCF7 cell line, similar to cervical carcinoma, indicates plausible site of rearrangement of viral genome [45].

Final validation of HPV16 occurrence in BC samples was done using Sanger sequencing of the LCR and E6/E7 region of the HPV16 genome. Additionally, sequence variation analysis revealed 7521G>A as the major (62.5%) sequence variant in the LCR region. This site overlapped with the binding of transcriptional repressor, YY1, which already been reported to regulate E6/E7 expression[46]. Other than the common ones, four novel variants in the LCR regions (7628A>T, 7800A>G, 7837A>G and 7839A>C) was also observed, which coincided with the predicted Oct-1/Pit-1a transcription factors binding sites [47]. In E6 locus, the major (70.0%) variant was 350T>G along with the familiar variants [48]. This 350T>G was also significantly associated with pathogenesis of HPV [49]. Moreover, *in silico* analysis revealed that the two novel variants 519G>A and 395G>T appears to destabilize the E6 mRNA and protein stability. In E7 gene, two observed synonymous sequence variation of 795T>G and 789T>C sites were already been reported in cervical carcinoma [25].

It is known that in cervical carcinoma AA lineage show higher virulence than other lineages [50]. But among Indian BC patients, E-G350 showed highest frequency (39.6%) followed by Ep (29.0%), NA1 (19.0%), NA1 A336G (8.0%), AA G395T (2.0%) and Af1 (2.0%). It is known that genome integration is associated with promoter hypomethylaltion [28]. Although majority of BC samples showed integration but comparatively higher methylation (96.7%) of P97 promoter and enhancer region (51.6%) was seen. On the contrary, predominant hypomethylation of P97 and enhancer region were seen in the integrated form of HPV16 in cervical carcinoma and head and neck squamous cell carcinoma (HNSCC) [28, 51]. Overall these results display significant differences in the pathogenesis process of HPV in BC, arising may be due to dissimilar tissue differentiation and microenvironment status. In addition, the absence of sequence variation in P97 promoter and enhancer sites indicates that the nucleotide variation may have occurred by other mechanism rather than DNA deamination after methylation. It appears that overall the mode of pathogenesis of HPV is different from that of cervical cancer.

Similar to other investigators, [16, 43] low copy number of HPV genome was seen in the present study among Indian BC patients. Likewise, such reports were present in non-genital cancers (like esophageal cancer and HNSCC) along with HPV16 positive cervical cancer cell line SiHa [52, 53]. This suggests that low viral load may be sufficient to promote carcinogenesis, especially if the viral DNA is integrated into the host genome [26]. Like cervical cancer, gradual increase in viral load with progression and severity of the disease was evident in the BC samples [22].

In our study, differential expression pattern of HPV16 E6, E7 and full length E6/E7 transcripts were seen. Though it was concordant with Lawson et al findings but, others author were unable to detect any E6 and E7 transcripts [14, 54]. This might be due to low copy number of viral genome and use of fresh tissue rather than paraffin embedded specimen used in previous analyses. Moreover, two spliced transcripts (E6*I and E6*II) of E6 and three spliced transcripts (E6*I/E7, E6*I/IE7, E6^E7) of E6/E7 were detected in the BC samples as previously seen in cervical carcinoma [25]. Additionally, two novel fusion transcripts E6^E7*I and E6^E7*II of E6/E7 were observed. Among these, donor and acceptor splicing sites of E6^E7*II transcript was previously predicted bioinformatically [55]. Lower stability of E6^E7*II than E6^E7*I transcripts but comparable half-life of its corresponding fusion proteins was further predicted *in silico*. Therefore, the identified HPV genome showed functional transcriptional activity in BC tissues. This was further concordant with the differential nuclear expression of E6 and E7 proteins in the BC samples similar to cervical carcinoma and HNSCC [14, 56].

**Fig 7:**
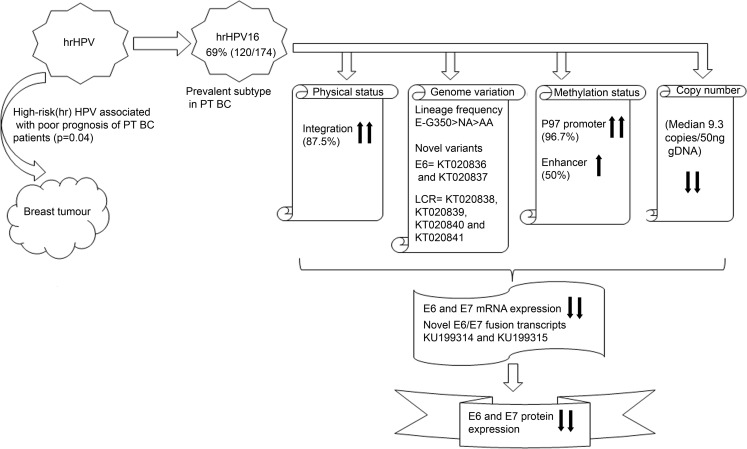
Schemetric diagram represent summerised results of the current study. Here, upper head arrow indicates high, lower head arrow indicates low. PT represent pre-therapeutic BC. gDNA; Genomic DNA of patients.

A sum up of results was provided to give an overall picture of genetic (physical status, genome variations and copy number) and epigenetic (methylation status) spectrum of HPV16 in breast tumors (Fig 7). Frequent infection of hrHPV in BC along with significant association of hrHPV with poor prognosis suggests that HPV may be associated with BC in Indian (Eastern) population. When compared with CACX, high integration of HPV16 was found similar. But low copy number, hypermethylated p97 promoter and enhancer along with low E6 and E7 expression and novel fusion transcripts of E6/E7 may provide a clue for different mode of pathogenesis of HPV16 in BC. Thus, these outcomes indicated plausible involvement of HPV in BC. However, more study is needed to completely understand the role of HPV infection in development of BC.

## Supporting information

S1 Table Distribution of samples and different clinico-pathological parameters of pre-therapeutic and neo-adjuvant chemotherapy treated (NACT) breast cancer (BC) patients. (DOC)

S2 Table Detail information of primers used. (DOCX)

S3 Table Status of HPV infection associated with different clinico-pathological parameters of the pre-therapeutic and neo-adjuvant chemotherapy treated (NACT) BC patients. (DOC)

S4 Table (**a**) Median value of HPV16 copy number in BC samples. **(b)** Distribution of HPV16 copy number according to grade, stage, age, median age of onset and HPV16 lineage.
(DOC)

S5 Table Concordance between mRNA and protein expression of E6 and E7 gene of HPV16. (DOC)

S1 Fig Utilization of pre-therapeutic and neo-adjuvant chemotherapy treated primary breast tumor samples. Schematic diagram represent work flow and distribution of samples in different experimental procedure. [‘N’ represent number of samples; IHC: immunohistochemistry]
(TIF)

S2 Fig Validation of presence of HPV16 and HPV18 subtypes in breast tumor and MCF7 cell line by southern blot. Corresponding autoradiograph of **(a)** HPV16 **(b)** HPV18 detection agorose gel in NACT and pre-therapeutic sample.[Here M: 100bp marker, NC (Negative control) represent with out DNA, +Ve (positive control) represent HPV16, HPV18 and HPV33 plasmid in their respective subtypes]
(TIF)

S3 Fig Kaplan–Meir 5-year survival probability curves with cumulative survival of breast cancer patients (BC) based on total HPV status. No statistically significant association was observed in survival probability of HPV infected **(a)** pre-therapeutuc and **(b)** NACT patients.
(TIF)

S4 Fig Characterization of different splice form of E6 and E6/E7 transcripts of HPV16 in pre-therapeutic breast tumor. Representative sequence chromatogram of **(a)** E6*II/E7 transcript showing junction of the splicing site (nt225/526) **(b)** E6^E7 transcript showing junction of the splicing site (nt226/742) **(c)** E6^E7*I transcript showing junction of the splicing site (nt174/718) **(d)** E6^E7 *II transcript showing junction of the splicing site (nt221/850). Novel splice form was shown in bold.
(TIF)

## Abbreviations

hrHPV: High risk HPV
BC: Breast Cancer
PT: Pre-therapeutic
NACT: Neo-adjuvant chemotherapy treated
P97: Early promoter
