# Supplementary material for: Study of association and molecular analysis of human papillomavirus in breast cancer of Indian patients: Clinical and prognostic implication
Source: PLoS One. 2017 Feb 28;12(2):e0172760. doi: 10.1371/journal.pone.0172760 (PMC5330495; doi:10.1371/journal.pone.0172760)
Supplement: S1 Table — (DOC) [file pone.0172760.s001.doc]

**Table S1:** Different clinico-pathological parameters of pre-therapeutic and neo-adjuvant chemotherapy treated (NACT) breast cancer (BC) patients.

|  | **Pre-therapeutic breast cancer**  **N=272** | **Neo-adjuvant chemotherapy treated (NACT) breast cancer**  **N=41** |
| --- | --- | --- |
|  | **Samples (%)** | **Samples (%)** |
| **Tumor stage** |  |  |
| Stage-I/II | 94 (34.5) | 9 (3.3) |
| Stage-III/IV | 174 (63.9) | 32 (11.7) |
| Not known | 4 (1.5) |  |
| **Histopathological Tumor Grade** |  |  |
| Grade-I | 33 (12.1) | 3 (7.3) |
| Grade-II | 143 (52.6) | 19 (46.3) |
| Grade-III | 88 (32.4) | 19 (46.3) |
| Not known | 8 (2.9) |  |
| **Lymph node metastasis** |  |  |
| Positive | 178 (65.9) | 27 (65.9) |
| Negative | 92 (34.1) | 14 (34.1) |
| Not known |  |  |
| **Median age of onset** |  |  |
| ≤45yrs | 153 (56.2) | 25 (60.9) |
| >45yrs | 119 (43.7) | 16 (39.0) |
| **Material status** |  |  |
| Married | 258 (94.9) | 40 (97.6) |
| Single | 9 (3.3) | 1 (2.4) |
| Widow | 5 (1.8) | 0 (0.0) |
| **Menopausal status** |  |  |
| Premenopausal (≤45yr) | 147 (54.9) | 24 (58.5) |
| Postmenopausal (>45yr) | 121 (45.1) | 17 (41.5) |
| Not known | 4 (1.4) | 0 (0.0) |
| **BC subtypes** |  |  |
| Luminal A | 38 (14.0) | 8 (19.5) |
| Luminal B | 83 (30.5) | 16 (39.0) |
| Her2+ positive | 84 (30.8) | 7 (17.1) |
| TNBC | 67 (24.6) | 10 (24.4) |
| **Estrogen Receptor (ER)** |  |  |
| Positive | 85 (31.3) | 17 (41.5) |
| Negative | 187 (68.8) | 24 (58.5) |
| **Progesterone Receptor (PR)** |  |  |
| Positive | 92 (33.8) | 18 (43.9) |
| Negative | 180 (66.2) | 23 (56.1) |
| **Her2 Receptor** |  |  |
| Positive | 165 (60.7) | 23 (56.1) |
| Negative | 107 (39.3) | 18 (43.9) |
| **Religion** |  |  |
| Hindu | 220 (80.9) | 36 (87.8) |
| Muslim | 51 (18.8) | 5 (12.2) |
| Christian | 1 (0.4) | 0 (0.0) |
| **Family History** |  |  |
| BC to BC | 12 (4.4) | 2 (4.9) |
| Other cancer to BC | 24 (8.8) | 5 (12.2) |
| History negative | 63 (23.2) | 22 (53.7) |
| Not known | 173 (63.6) | 12 (29.3) |
| **Histology** |  |  |
| ILC | 5 (1.8) | 0 (0.0) |
| DCIS | 11 (4.0) | 1 (2.4) |
| IDC | 256 (94.1) | 40 (97.6) |
| **Sex** |  |  |
| Male | 2 (0.7) | 0 (0.0) |
| Female | 270 (99.3) | 41 (100.0) |
| **Parity** |  |  |
| Nulliparous(0) | 18 (7.1) | 3 (7.3) |
| Parous (≤1-10) | 237 (92.9) | 37 (90.2) |
| Not known | 17 (6.2) | 1 (2.4) |

TNBC: Triple Negative Breast Cancer; ILC: Infiltrating Lobular Carcinoma; DICS: Ductal Carcinoma In-situ; IDC: Infiltrating ductal carcinoma; N: Number of samples; yrs: years.
