# Supplementary material for: Study of association and molecular analysis of human papillomavirus in breast cancer of Indian patients: Clinical and prognostic implication
Source: PLoS One. 2017 Feb 28;12(2):e0172760. doi: 10.1371/journal.pone.0172760 (PMC5330495; doi:10.1371/journal.pone.0172760)
Supplement: S2 Table — (DOCX) [file pone.0172760.s002.docx]

**Table S2:** Detail information of primers used.

| Name of the primer | Location of primer | Name of the analysis | Sequence of primers | PCR condition | Amplified region(bp) |
| --- | --- | --- | --- | --- | --- |
| MY09 | nt6584-7035 | Detection of HPV | 5’ GCMCAGGGWCATAAYAATGG 3’ | 2mM/55⁰C/35 cycle | 452 |
| MY11 |  |  | 5’ CGTCCMARRGGAWACTGATC 3’ |  |  |
| E6 F | nt 27-233 | Detection of HPV16 | 5’ AGGGCGTAACCGAAATCGG 3’ | 2mM/58⁰C/35 cycle | 206 |
| E6 R |  |  | 5’ CATATACCTCACGTCGCA 3’ |  |  |
| HPV18 LCR F | nt7465-7825 | Detection of HPV18 | 5’ CATATACCTCACGTCGCA 3’ | 2mM/56⁰C/35 cycle | 361 |
| HPV18 LCR R |  |  | 5’ CGGTTGCATAAACTATGTAT 3’ |  |  |
| E2A F | nt2738-3189 | Determination of physical status of HPV16 | 5’ CGAGGACAAGGAAAACGA 3’ | 2mM/55⁰C/35 cycle | 471 |
| E2A R |  |  | 5’ CTTGACCCTCTACCACAG 3’ |  |  |
| E2B F | nt3220-3599 | Determination of physical status of HPV16 | 5’ GGTTTATATTATGTTCATGAAGG 3’ | 2mM/53⁰C/ 35 cycle | 370 |
| E2B R |  |  | 5’ TATGGGTGTAGTGTTACTATTACA 3’ |  |  |
| E2C F | nt3596-3853 | Determination of physical status of HPV16 | 5’ GTAATAGTAACACTACACCCATA 3’ | 2mM/54⁰C/35 cycle | 279 |
| E2C R |  |  | 5’ GGATGCAGTATCAAGATTTG 3’ |  |  |
| E6-mRNA F | nt 103-572 | Detection of E6 mRNA | 5’ AATGTTTCAGGACCCACAGG 3’ | 1.5mM/58⁰C/40 cycle | 470 |
| E6-mRNA R |  |  | 5’ TCTCCATGCATGATTACAGCT 3’ |  |  |
| E7-mRNA F | nt562-875 | Detection of E7 mRNA | 5’ ATGCATGGAGATACACCTACATTG 3’ | 1.5mM/54⁰C/40 cycle | 314 |
| E7-mRNA R |  |  | 5’ GGATCAGCCATGGTAGATTATGG 3’ |  |  |
| E6-mRNA F | nt103-875 | Detection of E6 /E7 full length mRNA | 5’ AATGTTTCAGGACCCACAGG 3’ | 1.5mM/55⁰C/40 cycle | 773 |
| E7-mRNA R |  |  | 5’ GGATCAGCCATGGTAGATTATGG 3’ |  |  |
| E6 F | nt29-847 | Sequencing of E6 &E7 genes | 5’ AGGGCGTAACCGAAATCGG 3’ | 2mM/53⁰C/35 cycle | 818 |
| E7-mRNA R |  |  | 5’ GGATCAGCCATGGTAGATTATGG 3’ |  |  |
| LCR 16F | nt7291-115 | Sequencing of LCR region | 5’ GCTTGTGTAACTATTGTGTCA 3’ | 2mm/54⁰C/35 cycle | 733 |
| LCR 16R |  |  | 5’ GTCCTGAAACATTGCAGTTCT 3’ |  |  |
| β-2 microglobulin F |  | Expression control | 5' GTGCTCGCGCTACTCTCTCT 3' | 2mM/54⁰C/40 cycle | 153 |
| β-2 microglobulin R |  |  | 5' TCAATGTCGGATGGATGAAA 3' |  |  |

E2A=Aminoacid terminal of E2 gene; E2B=Hinge region of E2 gene; E2A=Carboxy terminal of E2 gene; LCR= Long Control Region
