# Supplementary material for: Study of association and molecular analysis of human papillomavirus in breast cancer of Indian patients: Clinical and prognostic implication
Source: PLoS One. 2017 Feb 28;12(2):e0172760. doi: 10.1371/journal.pone.0172760 (PMC5330495; doi:10.1371/journal.pone.0172760)
Supplement: S3 Table — (DOC) [file pone.0172760.s003.doc]

**Table S3:** Status of HPV infection associated with different clinico-pathological parameters of the pre-therapeutic and neo-adjuvant chemotherapy treated (NACT) BC patients

| **Clinical parameters** | **Pre-therapeutic** | **HPV positive (%)** | **HPV negative (%)** | **OR (95% CI)** | **P value** | **NACT** | **HPV positive (%)** | **HPV negative (%)** | **OR (95% CI)** | **P value** |
| --- | --- | --- | --- | --- | --- | --- | --- | --- | --- | --- |
| **Stage** |  |  |  |  |  |  |  |  |  |  |
| I/II | n=94 | 63 (67.0) | 31 (33.0) | 0.82 (0.48-1.39) | 0.47 | n=9 | 4 (44.4) | 5 (55.6) | 4.46 (0.93-21.20) | 0.04* |
| III/IV | n=174 | 109 (62.6) | 65 (37.4) |  |  | n=32 | 25 (78.9) | 7 (21.1) |  |  |
| Not known | n=4 | 2 (50.0) | 2 (50.0) |  |  |  |  |  |  |  |
| **Grade** |  |  |  |  |  |  |  |  |  |  |
| I | n=33 | 21 (63.6) | 12 (36.4) | NA | 0.86 | n=3 | 2 (66.7) | 1 (33.3) |  | 0.55 |
| II | n=143 | 93 (65.0) | 50 (35.0) |  |  | n=19 | 15 (78.9) | 4 (21.1) |  |  |
| III | n=88 | 54 (61.4) | 34 (38.6) |  |  | n=19 | 12 (63.2) | 7 (36.8) |  |  |
| Not known | n=8 | 6 (75.0) | 2 (25.0) |  |  |  |  |  |  |  |
| **Lymph Node metastasis** |  |  |  |  |  |  |  |  |  |  |
| Positive | n=178 | 113 (63.5) | 65 (36.5) | 0.92 (0.54-1.56) | 0.77 | n=27 | 20 (74.1) | 7 (25.9) | 1.58 (0.39-6.38) | 0.51 |
| Negative | n=92 | 60 (65.2) | 32 (34.8) |  |  | n=14 | 9 (64.3) | 5 (35.5) |  |  |
| Not known | n=2 | 1 (50.0) | 1 (50.0) |  |  |  |  |  |  |  |
| **Median age of Onset** |  |  |  |  |  |  |  |  |  |  |
| ≤45 yrs | n=153 | 107(69.9) | 46(30.1) | 1.80(1.09-2.97) | 0.02* | n=25 | 18 (72.0) | 7 (28.0) | 1.16 (0.29-4.60) | 0.82 |
| >45 yrs | n=119 | 67(56.3) | 52(43.7) |  |  | n=16 | 11 (68.8) | 5 (31.3) |  |  |
| **Material status** |  |  |  |  |  |  |  |  |  |  |
| Married | n=258 | 163 (63.2) | 95 (36.8) | NA | 0.28 | n=40 | 29 (72.5) | 11 (27.5) | NA | 0.11 |
| Single | n=9 | 8 (88.9) | 1 (11.1) |  |  | n=1 | 0 (0.0) | 1 (100.0) |  |  |
| Widow | n=5 | 3 (60.0) | 2 (40.0) |  |  | n=0 | 0 (0.0) | 0 (0.0) |  |  |
| **Menopausal status** |  |  |  |  |  |  |  |  |  |  |
| Pre (≤45yr) | n=147 | 96 (65.3) | 51 (34.7) | 1.15 (0.70-1.90) | 0.57 | n=24 | 16 (66.7) | 7 (33.3) | 0.61 (0.15-2.51) | 0.49 |
| Post (>45yr) | n=121 | 75 (62.0) | 46 (38.0) |  |  | n=17 | 13 (76.5) | 4 (23.5) |  |  |
| Not known | n=4 | 3 (75.0) | 1 (25.0) |  |  |  |  |  |  |  |
| **BC subtypes** |  |  |  |  |  |  |  |  |  |  |
| Luminal A | n=38 | 23 (60.5) | 15 (39.5) | NA | 0.26 | n=8 | 3 (37.5) | 5 (62.5) | NA | 0.13 |
| Luminal B | n=83 | 58 (69.9) | 25 (30.1) |  |  | n=16 | 13 (81.3) | 3 (18.8) |  |  |
| Her2+ positive | n=84 | 56 (66.6) | 28 (33.3) |  |  | n=7 | 5 (71.4) | 2 (28.6) |  |  |
| TNBC | n=67 | 37 (55.2) | 30 (44.8) |  |  | n=10 | 8 (80.0) | 2 20.0) |  |  |
| **Estrogen Receptor (ER)** |  |  |  |  |  |  |  |  |  |  |
| Positive | n=85 | 56 (65.9) | 29 (34.1) | 1.12 (0.65-1.93) | 0.65 | n=17 | 10 (58.8) | 7 (41.2) | 0.37 (0.09-1.49) | 0.15 |
| Negative | n=187 | 118 (63.1) | 69 (36.9) |  |  | n=24 | 19 (79.2) | 5 (20.8) |  |  |
| **Progesterone Receptor (PR)** |  |  |  |  |  |  |  |  |  |  |
| Positive | n=92 | 59 (64.1) | 33 (35.9) | 1.00 (0.59-1.70) | 0.96 | n=18 | 11 (61.1) | 7 (38.9) | 0.43 (0.11-1.71) | 0.23 |
| Negative | n=180 | 115 (63.9) | 65 (36.1) |  |  | n=23 | 18 (78.3) | 5 (21.7) |  |  |
| **Her2 Receptor** |  |  |  |  |  |  |  |  |  |  |
| Positive | n=165 | 112 (67.9) | 53 (32.1) | 1.53 (0.92-2.53) | 0.09 | n=23 | 18 (78.3) | 5 (21.7) | 2.29 (0.58-9.02) | 0.23 |
| Negative | n=107 | 62 (57.9) | 45 (42.1) |  |  | n=18 | 11 (61.1) | 7 (38.9) |  |  |
| **Religious** |  |  |  |  |  |  |  |  |  |  |
| Hindu | n=220 | 138 (62.7) | 82 (37.3) | NA | 0.55 | n=36 | 26 (72.2) | 10 (27.8) | 1.73(0.25-11.96) | 0.57 |
| Muslim | n=51 | 35 (68.6) | 16 (31.4) |  |  | n=5 | 3 (60.0) | 2 (40.0) |  |  |
| Christian | n=1 | 1 (100) | 0 (0.0) |  |  | n=0 | 0 (0.0) | 0 (0.0) |  |  |
| **Family History** |  |  |  |  |  |  |  |  |  |  |
| BC to BC | n=12 | 7 (58.3) | 5 (41.7) | NA | 0.08 | n=2 | 2 (100.0) | 0 (0.0) | NA | 0.73 |
| Other cancer to BC | n=24 | 17 (70.7) | 7 (29.2) |  |  | n=5 | 3 (60.0) | 2 (40.0) |  |  |
| History negative | n=63 | 48 (76.2) | 15 (23.8) |  |  | n=22 | 15 (68.2) | 7 (31.8) |  |  |
| Not known | n=173 | 102 (59.0) | 71 (41.0) |  |  | n=12 | 9 (75.0) | 3 (25.0) |  |  |
| **Histology** |  |  |  |  |  |  |  |  |  |  |
| ILC | n=5 | 4 (80.0) | 1 (20) | NA | 0.90 | n=0 | 4 (0.0) | 0 (0.0) | NA | 0.51 |
| DCIS | n=11 | 8 (72.7) | 3 (27.2) |  |  | n=1 | 1 (60.0) | 0 (0.0) |  |  |
| IDC | n=256 | 162 (63.3) | 94 (36.7) |  |  | n=40 | 28 (70.0) | 12 (30.0) |  |  |
| Not known | n=0 | 0 (0.0) | 0(0.0) |  |  |  |  |  |  |  |
| **Sex** |  |  |  |  |  |  |  |  |  |  |
| Male | n=2 | 2 (100) | 0 | NA | 0.40 | n=0 | 0 (0.0) | 0 (0.0) | NA | NA |
| Female | n=270 | 172 (63.7) | 98 (36.3) |  |  | n=41 | 29 (70.7) | 12 (29.3) |  |  |
| **parity** |  |  |  |  |  |  |  |  |  |  |
| Nulliparous(0) | n=18 | 15 (83.3) | 3 (16.7) | 0.33 (0.09-1.18) | 0.07 | n=3 | 1 (33.3) | 2 (66.7) | 5.40 (0.43-66.29) | 0.14 |
| Parous (≤1-10) | n=237 | 148 (62.4) | 89 (37.6) |  |  | n=37 | 27 (73.0) | 10 (27.0) |  |  |
| Not known | n=17 | 11 (64.7) | 6 (35.2) |  |  | n=1 | 1(100.0) | 0(0.0) |  |  |

TNBC: Triple Negative Breast Cancer; ILC: Infiltrating Lobular Carcinoma; DICS: Ductal Carcinoma In-situ; IDC: Infiltrating ductal carcinoma; NA: Not Applicable; “n” denotes sample number; Grade: Histopathological tumor grade; yrs: years; “ *” indicate significant correlation (P≤0.05); OR: Odds Ratio
