# Supplementary material for: Study of association and molecular analysis of human papillomavirus in breast cancer of Indian patients: Clinical and prognostic implication
Source: PLoS One. 2017 Feb 28;12(2):e0172760. doi: 10.1371/journal.pone.0172760 (PMC5330495; doi:10.1371/journal.pone.0172760)
Supplement: S4 Table — (a) Median value of HPV16 copy number in BC samples. (b) Distribution of HPV16 copy number according to grade, stage, age, median age of onset and HPV16 lineage. (DOC) [file pone.0172760.s004.doc]

**Table S4a**: Median value of HPV16 copy number in BC samples.

|  | **Median**  **(copies/50ng gDNA)** | **Range (Minimum-Maximum)**  **(copies/50ng gDNA)** |
| --- | --- | --- |
| Total BC | 9.3 | 0.58-1044.6 |

gDNA: Genomic DNA

**Table S4b:** Distribution of HPV16 copy number according to grade, stage, age, median age of onset and HPV16 lineage.

| **Parameters** | | **Median**  **(copies/50ng gDNA)** | **Range (Minimum-Maximum)**  **(copies/50ng gDNA)** |
| --- | --- | --- | --- |
| Grade | I(n=6) | 1.93 | 0.58-23.38 |
|  | II(n=13) | 2.87 | 1.14-57.4 |
|  | III(n=14) | 220.1 | 14.4-1044.6 |
| Stage | I/II(n=7) | 2.87 | 0.58-1044.6 |
|  | III/IV(n=26) | 23.38 | 1.22-952.7 |
| Median age of onset | ≤40yrs (n=17) | 14.4 | 0.58-952.7 |
|  | >40yrs(n=16) | 4.7 | 1-1044.6 |
| Lymph node metastasis | Positive (n=21) | 20.97 | 1.14-952.7 |
|  | Negative (n=12) | 1.79 | 0.58-1044.6 |
| HPV16 lineage | Ep (n=9) | 6.01 | 1-53.57 |
|  | E-G350 (n=17) | 57.4 | 0.58-1044.6 |
|  | NA1 (n=7) | 1.65 | 1.14-24.48 |

gDNA: Genomic DNA; Ep: European Prototype; E-G350: European T350G variant; NA1: North American 1; Grade: Histopathological tumour grade; yrs: years; n: number of samples
