# Supplementary material for: Study of association and molecular analysis of human papillomavirus in breast cancer of Indian patients: Clinical and prognostic implication
Source: PLoS One. 2017 Feb 28;12(2):e0172760. doi: 10.1371/journal.pone.0172760 (PMC5330495; doi:10.1371/journal.pone.0172760)
Supplement: S5 Table — (DOC) [file pone.0172760.s005.doc]

**Table S5:** Concordance between mRNA and protein expression of E6 and E7 gene of HPV16.

| **Gene name** | **High expression** | | **Low expression** | |
| --- | --- | --- | --- | --- |
|  | **mRNA (%)** | **Protein (%)** | **mRNA (%)** | **Protein (%)** |
| E6 (n=8) | 4 (50.0) | 4 (50.0) | 4 (50.0) | 4 (50.0) |
| E7 (n=8) | 4 (50.0) | 5 (62.5) | 4 (50.0) | 3 (37.5) |
| P value | 0.81 | | 0.78 | |

High/ moderate expression: High expression; “n” denotes number of sample
