# Supplementary figures and images for: Study of association and molecular analysis of human papillomavirus in breast cancer of Indian patients: Clinical and prognostic implication
Source: PLoS One. 2017 Feb 28;12(2):e0172760. doi: 10.1371/journal.pone.0172760 (PMC5330495; doi:10.1371/journal.pone.0172760)

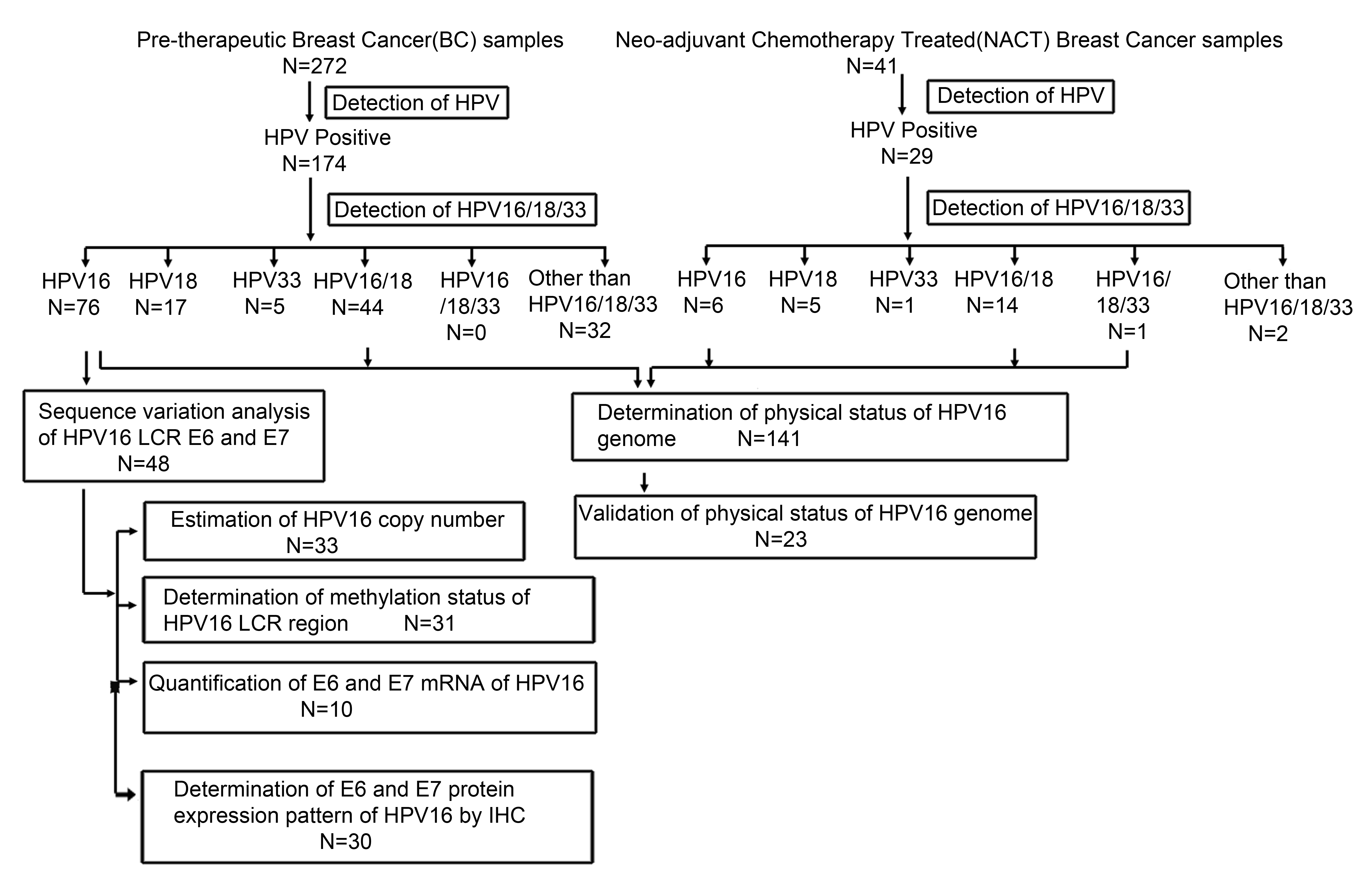

Supplement: S1 Fig — Schematic diagram represent work flow and distribution of samples in different experimental procedure. [‘N’ represent number of samples; IHC: immunohistochemistry] (TIF) [file pone.0172760.s006.tif]

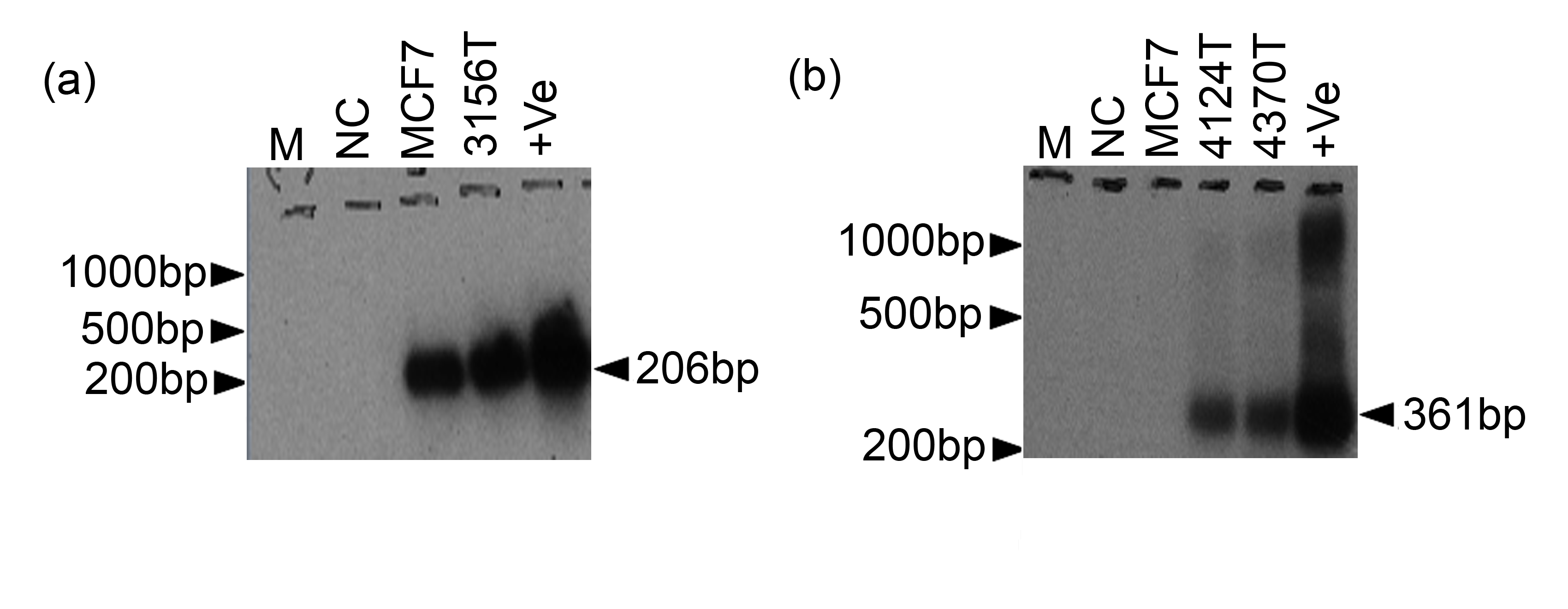

Supplement: S2 Fig — Corresponding autoradiograph of (a) HPV16 (b) HPV18 detection agorose gel in NACT and pre-therapeutic sample.[Here M: 100bp marker, NC (Negative control) represent with out DNA, +Ve (positive control) represent HPV16, HPV18 and HPV33 plasmid in their respective subtypes] (TIF) [file pone.0172760.s007.tif]

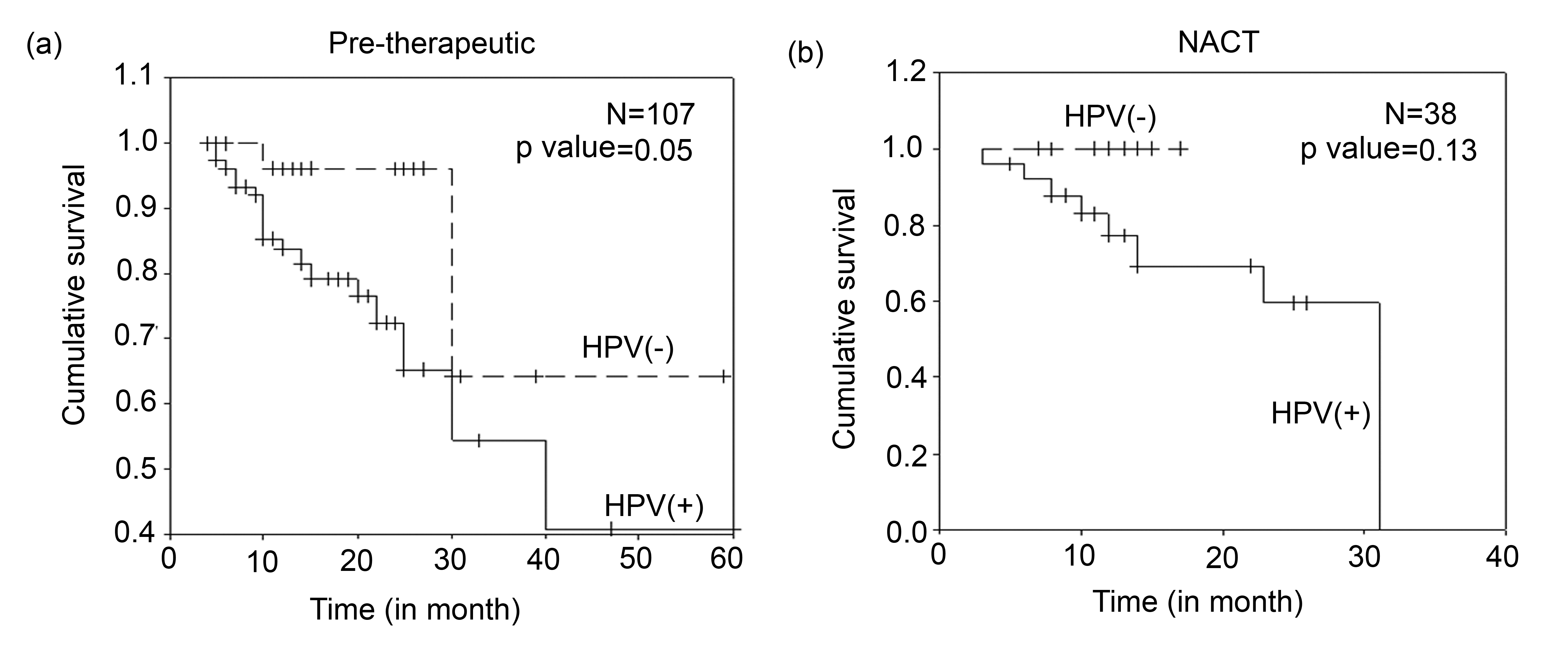

Supplement: S3 Fig — No statistically significant association was observed in survival probability of HPV infected (a) pre-therapeutuc and (b) NACT patients. (TIF) [file pone.0172760.s008.tif]

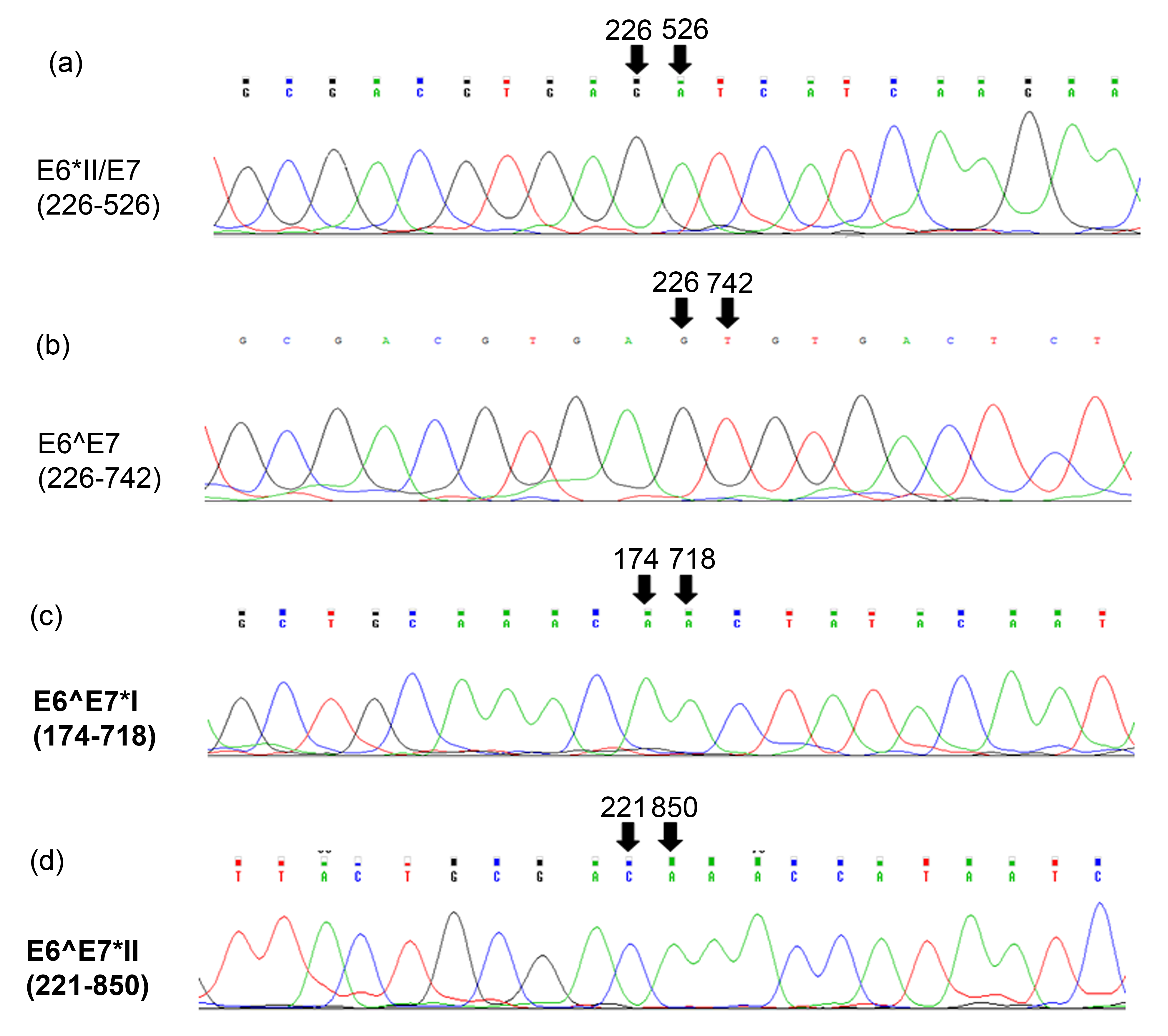

Supplement: S4 Fig — Representative sequence chromatogram of (a) E6*II/E7 transcript showing junction of the splicing site (nt225/526) (b) E6^E7 transcript showing junction of the splicing site (nt226/742) (c) E6^E7*I transcript showing junction of the splicing site (nt174/718) (d) E6^E7 *II transcript showing junction of the splicing site (nt221/850). Novel splice form was shown in bold. (TIF) [file pone.0172760.s009.tif]
